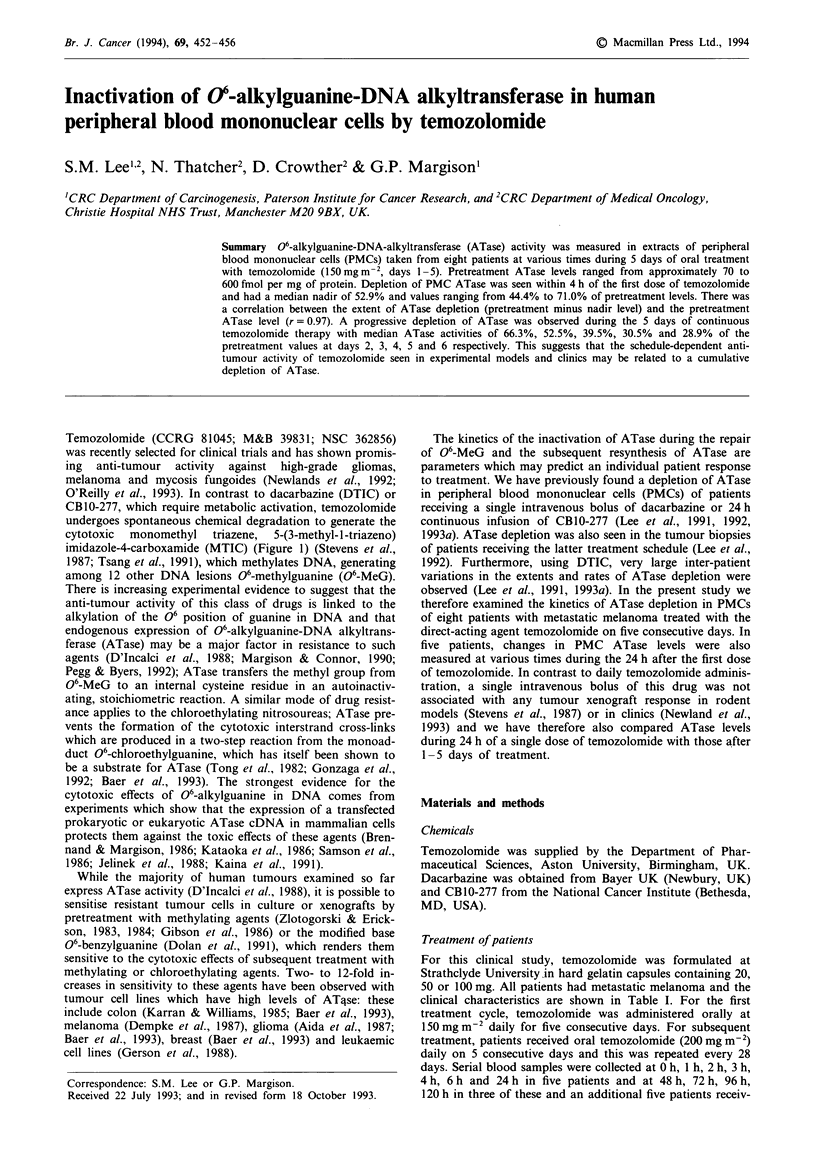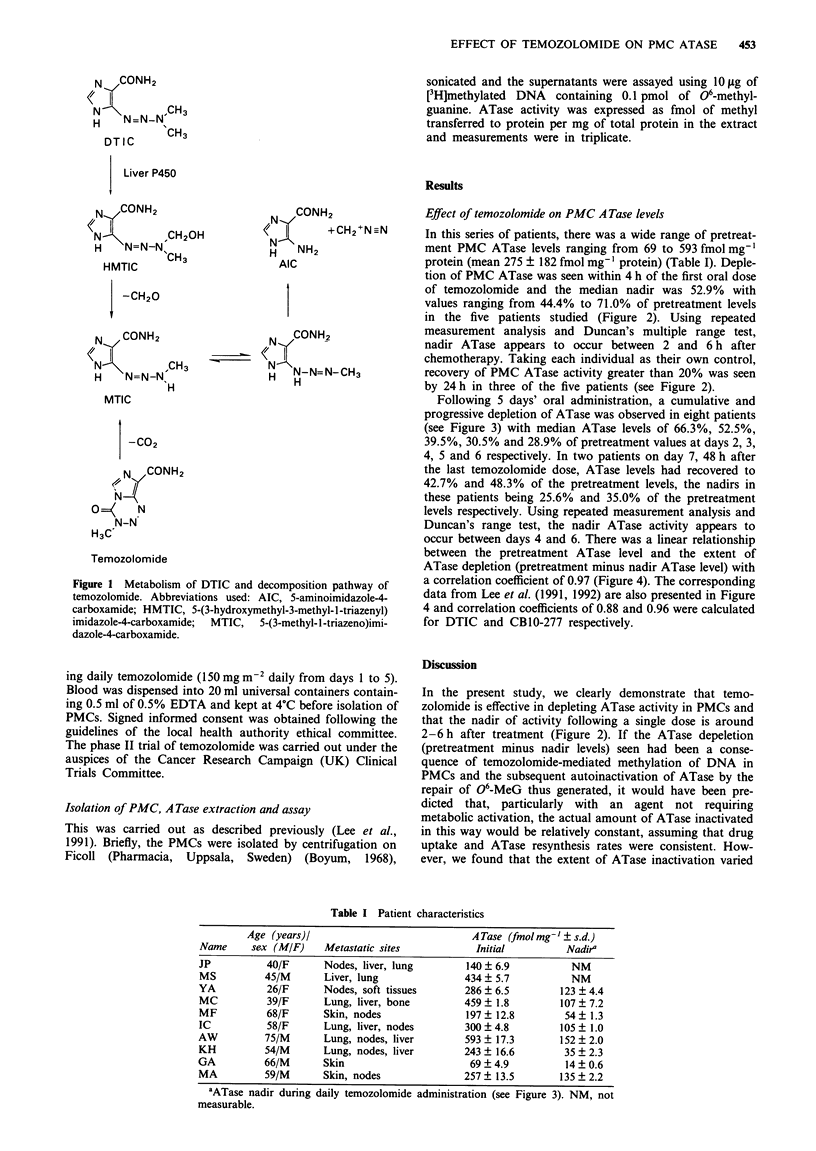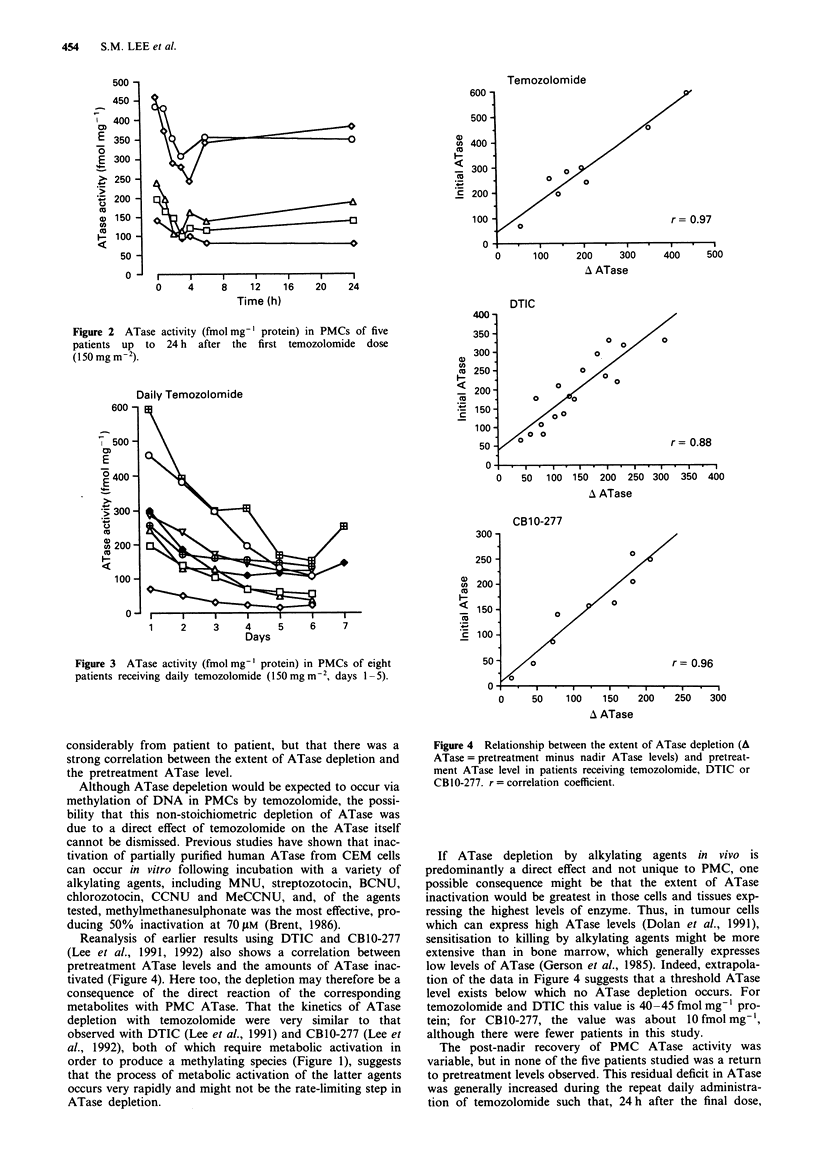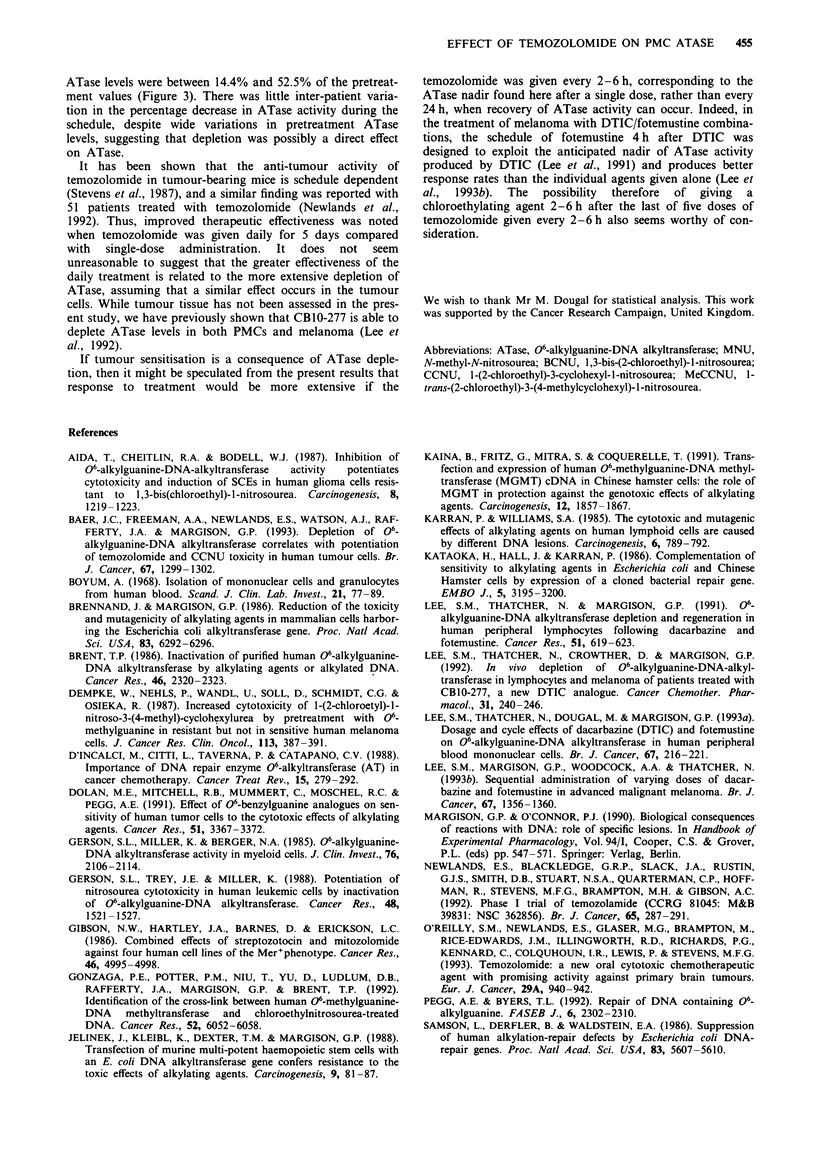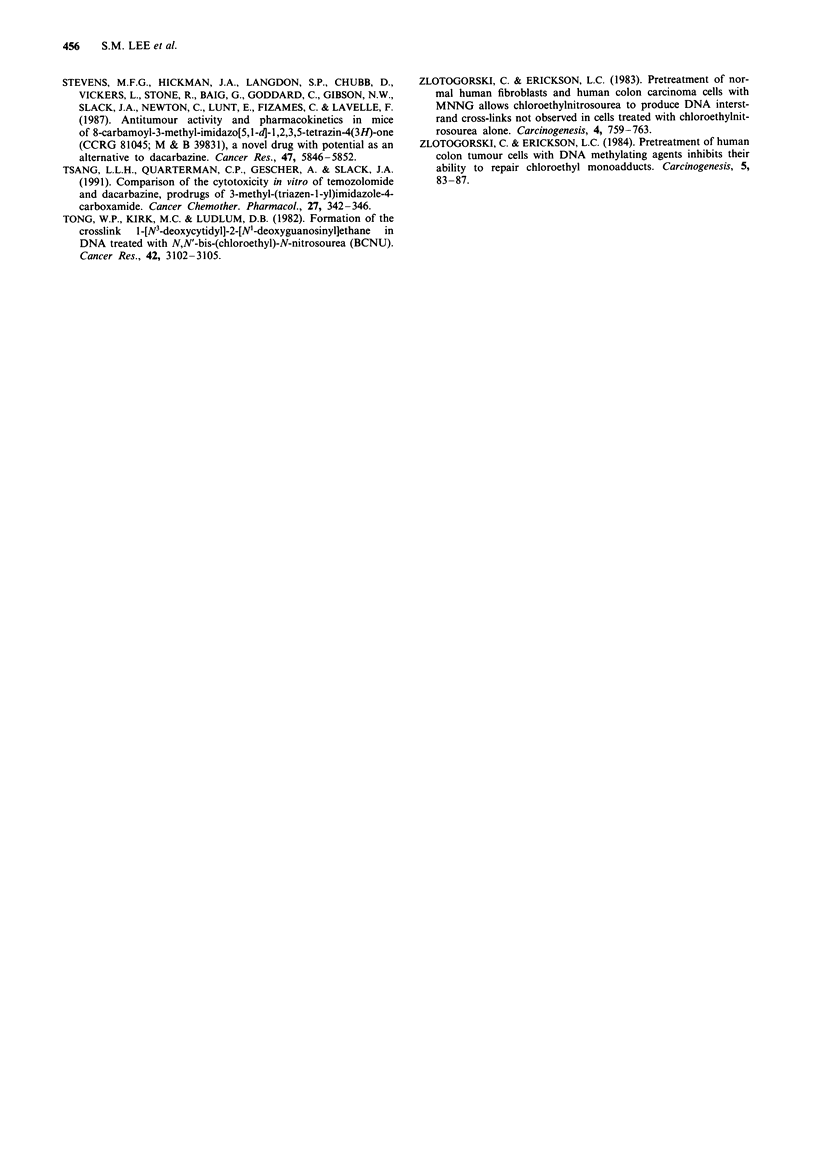# Inactivation of O6-alkylguanine-DNA alkyltransferase in human peripheral blood mononuclear cells by temozolomide.

**DOI:** 10.1038/bjc.1994.82

**Published:** 1994-03

**Authors:** S. M. Lee, N. Thatcher, D. Crowther, G. P. Margison

**Affiliations:** CRC Department of Carcinogenesis, Paterson Institute for Cancer Research, Christie Hospital NHS Trust, Manchester, UK.

## Abstract

O6-alkylguanine-DNA-alkyltransferase (ATase) activity was measured in extracts of peripheral blood mononuclear cells (PMCs) taken from eight patients at various times during 5 days of oral treatment with temozolomide (150 mg m-2, days 1-5). Pretreatment ATase levels ranged from approximately 70 to 600 fmol per mg of protein. Depletion of PMC ATase was seen within 4 h of the first dose of temozolomide and had a median nadir of 52.9% and values ranging from 44.4% to 71.0% of pretreatment levels. There was a correlation between the extent of ATase depletion (pretreatment minus nadir level) and the pretreatment ATase level (r = 0.97). A progressive depletion of ATase was observed during the 5 days of continuous temozolomide therapy with median ATase activities of 66.3%, 52.5%, 39.5%, 30.5% and 28.9% of the pretreatment values at days 2, 3, 4, 5 and 6 respectively. This suggests that the schedule-dependent anti-tumour activity of temozolomide seen in experimental models and clinics may be related to a cumulative depletion of ATase.


Br. J. Cancer (1994), 69, 452-456 © Macmillan Press Ltd., 1994

### Inactivation of $O^6$-alkylguanine-DNA alkyltransferase in human peripheral blood mononuclear cells by temozolomide

S.M. Lee[1,2], N. Thatcher[2], D. Crowther[2] & G.P. Margison[1]

[1]CRC Department of Carcinogenesis, Paterson Institute for Cancer Research, and [2]CRC Department of Medical Oncology, Christie Hospital NHS Trust, Manchester M20 9BX, UK.

**Summary** $O^6$-alkylguanine-DNA-alkyltransferase (ATase) activity was measured in extracts of peripheral blood mononuclear cells (PMCs) taken from eight patients at various times during 5 days of oral treatment with temozolomide (150 mg m$^{-2}$, days 1-5). Pretreatment ATase levels ranged from approximately 70 to 600 fmol per mg of protein. Depletion of PMC ATase was seen within 4 h of the first dose of temozolomide and had a median nadir of 52.9% and values ranging from 44.4% to 71.0% of pretreatment levels. There was a correlation between the extent of ATase depletion (pretreatment minus nadir level) and the pretreatment ATase level ($r = 0.97$). A progressive depletion of ATase was observed during the 5 days of continuous temozolomide therapy with median ATase activities of 66.3%, 52.5%, 39.5%, 30.5% and 28.9% of the pretreatment values at days 2, 3, 4, 5 and 6 respectively. This suggests that the schedule-dependent anti-tumour activity of temozolomide seen in experimental models and clinics may be related to a cumulative depletion of ATase.

Temozolomide (CCRG 81045; M&B 39831; NSC 362856) was recently selected for clinical trials and has shown promising anti-tumour activity against high-grade gliomas, melanoma and mycosis fungoides (Newlands *et al.*, 1992; O'Reilly *et al.*, 1993). In contrast to dacarbazine (DTIC) or CB10-277, which require metabolic activation, temozolomide undergoes spontaneous chemical degradation to generate the cytotoxic monomethyl triazene, 5-(3-methyl-1-triazeno) imidazole-4-carboxamide (MTIC) (Figure 1) (Stevens *et al.*, 1987; Tsang *et al.*, 1991), which methylates DNA, generating among 12 other DNA lesions $O^6$-methylguanine ($O^6$-MeG). There is increasing experimental evidence to suggest that the anti-tumour activity of this class of drugs is linked to the alkylation of the $O^6$ position of guanine in DNA and that endogenous expression of $O^6$-alkylguanine-DNA alkyltransferase (ATase) may be a major factor in resistance to such agents (D'Incalci *et al.*, 1988; Margison & Connor, 1990; Pegg & Byers, 1992); ATase transfers the methyl group from $O^6$-MeG to an internal cysteine residue in an autoinactivating, stoichiometric reaction. A similar mode of drug resistance applies to the chloroethylating nitrosoureas; ATase prevents the formation of the cytotoxic interstrand cross-links which are produced in a two-step reaction from the monoadduct $O^6$-chloroethylguanine, which has itself been shown to be a substrate for ATase (Tong *et al.*, 1982; Gonzaga *et al.*, 1992; Baer *et al.*, 1993). The strongest evidence for the cytotoxic effects of $O^6$-alkylguanine in DNA comes from experiments which show that the expression of a transfected prokaryotic or eukaryotic ATase cDNA in mammalian cells protects them against the toxic effects of these agents (Brennand & Margison, 1986; Kataoka *et al.*, 1986; Samson *et al.*, 1986; Jelinek *et al.*, 1988; Kaina *et al.*, 1991).

While the majority of human tumours examined so far express ATase activity (D'Incalci *et al.*, 1988), it is possible to sensitise resistant tumour cells in culture or xenografts by pretreatment with methylating agents (Zlotogorski & Erickson, 1983, 1984; Gibson *et al.*, 1986) or the modified base $O^6$-benzylguanine (Dolan *et al.*, 1991), which renders them sensitive to the cytotoxic effects of subsequent treatment with methylating or chloroethylating agents. Two- to 12-fold increases in sensitivity to these agents have been observed with tumour cell lines which have high levels of ATase: these include colon (Karran & Williams, 1985; Baer *et al.*, 1993), melanoma (Dempke *et al.*, 1987), glioma (Aida *et al.*, 1987; Baer *et al.*, 1993), breast (Baer *et al.*, 1993) and leukaemic cell lines (Gerson *et al.*, 1988).

Correspondence: S.M. Lee or G.P. Margison.
Received 22 July 1993; and in revised form 18 October 1993.

The kinetics of the inactivation of ATase during the repair of $O^6$-MeG and the subsequent resynthesis of ATase are parameters which may predict an individual patient response to treatment. We have previously found a depletion of ATase in peripheral blood mononuclear cells (PMCs) of patients receiving a single intravenous bolus of dacarbazine or 24 h continuous infusion of CB10-277 (Lee *et al.*, 1991, 1992, 1993*a*). ATase depletion was also seen in the tumour biopsies of patients receiving the latter treatment schedule (Lee *et al.*, 1992). Furthermore, using DTIC, very large inter-patient variations in the extents and rates of ATase depletion were observed (Lee *et al.*, 1991, 1993*a*). In the present study we therefore examined the kinetics of ATase depletion in PMCs of eight patients with metastatic melanoma treated with the direct-acting agent temozolomide on five consecutive days. In five patients, changes in PMC ATase levels were also measured at various times during the 24 h after the first dose of temozolomide. In contrast to daily temozolomide administration, a single intravenous bolus of this drug was not associated with any tumour xenograft response in rodent models (Stevens *et al.*, 1987) or in clinics (Newland *et al.*, 1993) and we have therefore also compared ATase levels during 24 h of a single dose of temozolomide with those after 1-5 days of treatment.

### Materials and methods

### *Chemicals*

Temozolomide was supplied by the Department of Pharmaceutical Sciences, Aston University, Birmingham, UK. Dacarbazine was obtained from Bayer UK (Newbury, UK) and CB10-277 from the National Cancer Institute (Bethesda, MD, USA).

### *Treatment of patients*

For this clinical study, temozolomide was formulated at Strathclyde University in hard gelatin capsules containing 20, 50 or 100 mg. All patients had metastatic melanoma and the clinical characteristics are shown in Table I. For the first treatment cycle, temozolomide was administered orally at 150 mg m$^{-2}$ daily for five consecutive days. For subsequent treatment, patients received oral temozolomide (200 mg m$^{-2}$) daily on 5 consecutive days and this was repeated every 28 days. Serial blood samples were collected at 0 h, 1 h, 2 h, 3 h, 4 h, 6 h and 24 h in five patients and at 48 h, 72 h, 96 h, 120 h in three of these and an additional five patients receiv-

**Figure 1** Metabolism of DTIC and decomposition pathway of temozolomide. Abbreviations used: AIC, 5-aminoimidazole-4-carboxamide; HMTIC, 5-(3-hydroxymethyl-3-methyl-1-triazenyl) imidazole-4-carboxamide; MTIC, 5-(3-methyl-1-triazeno)imidazole-4-carboxamide.

ing daily temozolomide (150 mg $m^{-2}$ daily from days 1 to 5). Blood was dispensed into 20 ml universal containers containing 0.5 ml of 0.5% EDTA and kept at 4°C before isolation of PMCs. Signed informed consent was obtained following the guidelines of the local health authority ethical committee. The phase II trial of temozolomide was carried out under the auspices of the Cancer Research Campaign (UK) Clinical Trials Committee.

*Isolation of PMC, ATase extraction and assay*

This was carried out as described previously (Lee *et al.*, 1991). Briefly, the PMCs were isolated by centrifugation on Ficoll (Pharmacia, Uppsala, Sweden) (Boyum, 1968), sonicated and the supernatants were assayed using 10 µg of [$^3$H]methylated DNA containing 0.1 pmol of $O^6$-methylguanine. ATase activity was expressed as fmol of methyl transferred to protein per mg of total protein in the extract and measurements were in triplicate.

### Results

*Effect of temozolomide on PMC ATase levels*

In this series of patients, there was a wide range of pretreatment PMC ATase levels ranging from 69 to 593 fmol $mg^{-1}$ protein (mean 275 ± 182 fmol $mg^{-1}$ protein) (Table I). Depletion of PMC ATase was seen within 4 h of the first oral dose of temozolomide and the median nadir was 52.9% with values ranging from 44.4% to 71.0% of pretreatment levels in the five patients studied (Figure 2). Using repeated measurement analysis and Duncan's multiple range test, nadir ATase appears to occur between 2 and 6 h after chemotherapy. Taking each individual as their own control, recovery of PMC ATase activity greater than 20% was seen by 24 h in three of the five patients (see Figure 2).

Following 5 days' oral administration, a cumulative and progressive depletion of ATase was observed in eight patients (see Figure 3) with median ATase levels of 66.3%, 52.5%, 39.5%, 30.5% and 28.9% of pretreatment values at days 2, 3, 4, 5 and 6 respectively. In two patients on day 7, 48 h after the last temozolomide dose, ATase levels had recovered to 42.7% and 48.3% of the pretreatment levels, the nadirs in these patients being 25.6% and 35.0% of the pretreatment levels respectively. Using repeated measurement analysis and Duncan's range test, the nadir ATase activity appears to occur between days 4 and 6. There was a linear relationship between the pretreatment ATase level and the extent of ATase depletion (pretreatment minus nadir ATase level) with a correlation coefficient of 0.97 (Figure 4). The corresponding data from Lee *et al.* (1991, 1992) are also presented in Figure 4 and correlation coefficients of 0.88 and 0.96 were calculated for DTIC and CB10-277 respectively.

### Discussion

In the present study, we clearly demonstrate that temozolomide is effective in depleting ATase activity in PMCs and that the nadir of activity following a single dose is around 2–6 h after treatment (Figure 2). If the ATase depeletion (pretreatment minus nadir levels) seen had been a consequence of temozolomide-mediated methylation of DNA in PMCs and the subsequent autoinactivation of ATase by the repair of $O^6$-MeG thus generated, it would have been predicted that, particularly with an agent not requiring metabolic activation, the actual amount of ATase inactivated in this way would be relatively constant, assuming that drug uptake and ATase resynthesis rates were consistent. However, we found that the extent of ATase inactivation varied

**Table I** Patient characteristics

| Name | Age (years)/ sex (M/F) | Metastatic sites | ATase (fmol $mg^{-1}$ ± s.d.) Initial | Nadir[a] |
|---|---|---|---|---|
| JP | 40/F | Nodes, liver, lung | 140 ± 6.9 | NM |
| MS | 45/M | Liver, lung | 434 ± 5.7 | NM |
| YA | 26/F | Nodes, soft tissues | 286 ± 6.5 | 123 ± 4.4 |
| MC | 39/F | Lung, liver, bone | 459 ± 1.8 | 107 ± 7.2 |
| MF | 68/F | Skin, nodes | 197 ± 12.8 | 54 ± 1.3 |
| IC | 58/F | Lung, liver, nodes | 300 ± 4.8 | 105 ± 1.0 |
| AW | 75/M | Lung, nodes, liver | 593 ± 17.3 | 152 ± 2.0 |
| KH | 54/M | Lung, nodes, liver | 243 ± 16.6 | 35 ± 2.3 |
| GA | 66/M | Skin | 69 ± 4.9 | 14 ± 0.6 |
| MA | 59/M | Skin, nodes | 257 ± 13.5 | 135 ± 2.2 |

[a]ATase nadir during daily temozolomide administration (see Figure 3). NM, not measurable.

Figure 2 ATase activity (fmol mg$^{-1}$ protein) in PMCs of five patients up to 24 h after the first temozolomide dose (150 mg m$^{-2}$).

Figure 3 ATase activity (fmol mg$^{-1}$ protein) in PMCs of eight patients receiving daily temozolomide (150 mg m$^{-2}$, days 1–5).

Figure 4 Relationship between the extent of ATase depletion (Δ ATase = pretreatment minus nadir ATase levels) and pretreatment ATase level in patients receiving temozolomide, DTIC or CB10-277. r = correlation coefficient.

considerably from patient to patient, but that there was a strong correlation between the extent of ATase depletion and the pretreatment ATase level.

Although ATase depeletion would be expected to occur via methylation of DNA in PMCs by temozolomide, the possibility that this non-stoichiometric depletion of ATase was due to a direct effect of temozolomide on the ATase itself cannot be dismissed. Previous studies have shown that inactivation of partially purified human ATase from CEM cells can occur *in vitro* following incubation with a variety of alkylating agents, including MNU, streptozotocin, BCNU, chlorozotocin, CCNU and MeCCNU, and, of the agents tested, methylmethanesulphonate was the most effective, producing 50% inactivation at 70 $\mu$M (Brent, 1986).

Reanalysis of earlier results using DTIC and CB10-277 (Lee *et al.*, 1991, 1992) also shows a correlation between pretreatment ATase levels and the amounts of ATase inactivated (Figure 4). Here too, the depletion may therefore be a consequence of the direct reaction of the corresponding metabolites with PMC ATase. That the kinetics of ATase depletion with temozolomide were very similar to that observed with DTIC (Lee *et al.*, 1991) and CB10-277 (Lee *et al.*, 1992), both of which require metabolic activation in order to produce a methylating species (Figure 1), suggests that the process of metabolic activation of the latter agents occurs very rapidly and might not be the rate-limiting step in ATase depletion.

If ATase depletion by alkylating agents *in vivo* is predominantly a direct effect and not unique to PMC, one possible consequence might be that the extent of ATase inactivation would be greatest in those cells and tissues expressing the highest levels of enzyme. Thus, in tumour cells which can express high ATase levels (Dolan *et al.*, 1991), sensitisation to killing by alkylating agents might be more extensive than in bone marrow, which generally expresses low levels of ATase (Gerson *et al.*, 1985). Indeed, extrapolation of the data in Figure 4 suggests that a threshold ATase level exists below which no ATase depletion occurs. For temozolomide and DTIC this value is 40–45 fmol mg$^{-1}$ protein; for CB10-277, the value was about 10 fmol mg$^{-1}$, although there were fewer patients in this study.

The post-nadir recovery of PMC ATase activity was variable, but in none of the five patients studied was a return to pretreatment levels observed. This residual deficit in ATase was generally increased during the repeat daily administration of temozolomide such that, 24 h after the final dose,

ATase levels were between 14.4% and 52.5% of the pretreatment values (Figure 3). There was little inter-patient variation in the percentage decrease in ATase activity during the schedule, despite wide variations in pretreatment ATase levels, suggesting that depletion was possibly a direct effect on ATase.

It has been shown that the anti-tumour activity of temozolomide in tumour-bearing mice is schedule dependent (Stevens *et al.*, 1987), and a similar finding was reported with 51 patients treated with temozolomide (Newlands *et al.*, 1992). Thus, improved therapeutic effectiveness was noted when temozolomide was given daily for 5 days compared with single-dose administration. It does not seem unreasonable to suggest that the greater effectiveness of the daily treatment is related to the more extensive depletion of ATase, assuming that a similar effect occurs in the tumour cells. While tumour tissue has not been assessed in the present study, we have previously shown that CB10-277 is able to deplete ATase levels in both PMCs and melanoma (Lee *et al.*, 1992).

If tumour sensitisation is a consequence of ATase depletion, then it might be speculated from the present results that response to treatment would be more extensive if the temozolomide was given every 2–6 h, corresponding to the ATase nadir found here after a single dose, rather than every 24 h, when recovery of ATase activity can occur. Indeed, in the treatment of melanoma with DTIC/fotemustine combinations, the schedule of fotemustine 4 h after DTIC was designed to exploit the anticipated nadir of ATase activity produced by DTIC (Lee *et al.*, 1991) and produces better response rates than the individual agents given alone (Lee *et al.*, 1993*b*). The possibility therefore of giving a chloroethylating agent 2–6 h after the last of five doses of temozolomide given every 2–6 h also seems worthy of consideration.

We wish to thank Mr M. Dougal for statistical analysis. This work was supported by the Cancer Research Campaign, United Kingdom.

Abbreviations: ATase, $O^6$-alkylguanine-DNA alkyltransferase; MNU, *N*-methyl-*N*-nitrosourea; BCNU, 1,3-bis-(2-chloroethyl)-1-nitrosourea; CCNU, 1-(2-chloroethyl)-3-cyclohexyl-1-nitrosourea; MeCCNU, 1-*trans*-(2-chloroethyl)-3-(4-methylcyclohexyl)-1-nitrosourea.

### References

AIDA, T., CHEITLIN, R.A. & BODELL, W.J. (1987). Inhibition of $O^6$-alkylguanine-DNA-alkyltransferase activity potentiates cytotoxicity and induction of SCEs in human glioma cells resistant to 1,3-bis(chloroethyl)-1-nitrosourea. *Carcinogenesis*, **8**, 1219–1223.

BAER, J.C., FREEMAN, A.A., NEWLANDS, E.S., WATSON, A.J., RAFFERTY, J.A. & MARGISON, G.P. (1993). Depletion of $O^6$-alkylguanine-DNA alkyltransferase correlates with potentiation of temozolomide and CCNU toxicity in human tumour cells. *Br. J. Cancer*, **67**, 1299–1302.

BOYUM, A. (1968). Isolation of mononuclear cells and granulocytes from human blood. *Scand. J. Clin. Lab. Invest.*, **21**, 77–89.

BRENNAND, J. & MARGISON, G.P. (1986). Reduction of the toxicity and mutagenicity of alkylating agents in mammalian cells harboring the Escherichia coli alkyltransferase gene. *Proc. Natl Acad. Sci. USA*, **83**, 6292–6296.

BRENT, T.P. (1986). Inactivation of purified human $O^6$-alkylguanine-DNA alkyltransferase by alkylating agents or alkylated DNA. *Cancer Res.*, **46**, 2320–2323.

DEMPKE, W., NEHLS, P., WANDL, U., SOLL, D., SCHMIDT, C.G. & OSIEKA, R. (1987). Increased cytotoxicity of 1-(2-chloroetyl)-1-nitroso-3-(4-methyl)-cyclohexylurea by pretreatment with $O^6$-methylguanine in resistant but not in sensitive human melanoma cells. *J. Cancer Res. Clin. Oncol.*, **113**, 387–391.

D'INCALCI, M., CITTI, L., TAVERNA, P. & CATAPANO, C.V. (1988). Importance of DNA repair enzyme $O^6$-alkyltransferase (AT) in cancer chemotherapy. *Cancer Treat Rev.*, **15**, 279–292.

DOLAN, M.E., MITCHELL, R.B., MUMMERT, C., MOSCHEL, R.C. & PEGG, A.E. (1991). Effect of $O^6$-benzylguanine analogues on sensitivity of human tumor cells to the cytotoxic effects of alkylating agents. *Cancer Res.*, **51**, 3367–3372.

GERSON, S.L., MILLER, K. & BERGER, N.A. (1985). $O^6$-alkylguanine-DNA alkyltransferase activity in myeloid cells. *J. Clin. Invest.*, **76**, 2106–2114.

GERSON, S.L., TREY, J.E. & MILLER, K. (1988). Potentiation of nitrosourea cytotoxicity in human leukemic cells by inactivation of $O^6$-alkylguanine-DNA alkyltransferase. *Cancer Res.*, **48**, 1521–1527.

GIBSON, N.W., HARTLEY, J.A., BARNES, D. & ERICKSON, L.C. (1986). Combined effects of streptozotocin and mitozolomide against four human cell lines of the $Mer^+$phenotype. *Cancer Res.*, **46**, 4995–4998.

GONZAGA, P.E., POTTER, P.M., NIU, T., YU, D., LUDLUM, D.B., RAFFERTY, J.A., MARGISON, G.P. & BRENT, T.P. (1992). Identification of the cross-link between human $O^6$-methylguanine-DNA methyltransferase and chloroethylnitrosourea-treated DNA. *Cancer Res.*, **52**, 6052–6058.

JELINEK, J., KLEIBL, K., DEXTER, T.M. & MARGISON, G.P. (1988). Transfection of murine multi-potent haemopoietic stem cells with an *E. coli* DNA alkyltransferase gene confers resistance to the toxic effects of alkylating agents. *Carcinogenesis*, **9**, 81–87.

KAINA, B., FRITZ, G., MITRA, S. & COQUERELLE, T. (1991). Transfection and expression of human $O^6$-methylguanine-DNA methyltransferase (MGMT) cDNA in Chinese hamster cells: the role of MGMT in protection against the genotoxic effects of alkylating agents. *Carcinogenesis*, **12**, 1857–1867.

KARRAN, P. & WILLIAMS, S.A. (1985). The cytotoxic and mutagenic effects of alkylating agents on human lymphoid cells are caused by different DNA lesions. *Carcinogenesis*, **6**, 789–792.

KATAOKA, H., HALL, J. & KARRAN, P. (1986). Complementation of sensitivity to alkylating agents in *Escherichia coli* and Chinese Hamster cells by expression of a cloned bacterial repair gene. *EMBO J.*, **5**, 3195–3200.

LEE, S.M., THATCHER, N. & MARGISON, G.P. (1991). $O^6$-alkylguanine-DNA alkyltransferase depletion and regeneration in human peripheral lymphocytes following dacarbazine and fotemustine. *Cancer Res.*, **51**, 619–623.

LEE, S.M., THATCHER, N., CROWTHER, D. & MARGISON, G.P. (1992). *In vivo* depletion of $O^6$-alkylguanine-DNA-alkyltransferase in lymphocytes and melanoma of patients treated with CB10-277, a new DTIC analogue. *Cancer Chemother. Pharmacol.*, **31**, 240–246.

LEE, S.M., THATCHER, N., DOUGAL, M. & MARGISON, G.P. (1993*a*). Dosage and cycle effects of dacarbazine (DTIC) and fotemustine on $O^6$-alkylguanine-DNA alkyltransferase in human peripheral blood mononuclear cells. *Br. J. Cancer*, **67**, 216–221.

LEE, S.M., MARGISON, G.P., WOODCOCK, A.A. & THATCHER, N. (1993*b*). Sequential administration of varying doses of dacarbazine and fotemustine in advanced malignant melanoma. *Br. J. Cancer*, **67**, 1356–1360.

MARGISON, G.P. & O'CONNOR, P.J. (1990). Biological consequences of reactions with DNA: role of specific lesions. In *Handbook of Experimental Pharmacology*, Vol. 94/I, Cooper, C.S. & Grover, P.L. (eds) pp. 547–571. Springer: Verlag, Berlin.

NEWLANDS, E.S., BLACKLEDGE, G.R.P., SLACK, J.A., RUSTIN, G.J.S., SMITH, D.B., STUART, N.S.A., QUARTERMAN, C.P., HOFFMAN, R., STEVENS, M.F.G., BRAMPTON, M.H. & GIBSON, A.C. (1992). Phase I trial of temozolamide (CCRG 81045: M&B 39831: NSC 362856). *Br. J. Cancer*, **65**, 287–291.

O'REILLY, S.M., NEWLANDS, E.S., GLASER, M.G., BRAMPTON, M., RICE-EDWARDS, J.M., ILLINGWORTH, R.D., RICHARDS, P.G., KENNARD, C., COLQUHOUN, I.R., LEWIS, P. & STEVENS, M.F.G. (1993). Temozolomide: a new oral cytotoxic chemotherapeutic agent with promising activity against primary brain tumours. *Eur. J. Cancer*, **29A**, 940–942.

PEGG, A.E. & BYERS, T.L. (1992). Repair of DNA containing $O^6$-alkylguanine. *FASEB J.*, **6**, 2302–2310.

SAMSON, L., DERFLER, B. & WALDSTEIN, E.A. (1986). Suppression of human alkylation-repair defects by *Escherichia coli* DNA-repair genes. *Proc. Natl Acad. Sci. USA*, **83**, 5607–5610.

STEVENS, M.F.G., HICKMAN, J.A., LANGDON, S.P., CHUBB, D., VICKERS, L., STONE, R., BAIG, G., GODDARD, C., GIBSON, N.W., SLACK, J.A., NEWTON, C., LUNT, E., FIZAMES, C. & LAVELLE, F. (1987). Antitumour activity and pharmacokinetics in mice of 8-carbamoyl-3-methyl-imidazo[5,1-*d*]-1,2,3,5-tetrazin-4(3*H*)-one (CCRG 81045; M & B 39831), a novel drug with potential as an alternative to dacarbazine. *Cancer Res.*, **47**, 5846–5852.

TSANG, L.L.H., QUARTERMAN, C.P., GESCHER, A. & SLACK, J.A. (1991). Comparison of the cytotoxicity *in vitro* of temozolomide and dacarbazine, prodrugs of 3-methyl-(triazen-1-yl)imidazole-4-carboxamide. *Cancer Chemother. Pharmacol.*, **27**, 342–346.

TONG, W.P., KIRK, M.C. & LUDLUM, D.B. (1982). Formation of the crosslink 1-[$N^3$-deoxycytidyl]-2-[$N^1$-deoxyguanosinyl]ethane in DNA treated with *N,N'*-bis-(chloroethyl)-*N*-nitrosourea (BCNU). *Cancer Res.*, **42**, 3102–3105.

ZLOTOGORSKI, C. & ERICKSON, L.C. (1983). Pretreatment of normal human fibroblasts and human colon carcinoma cells with MNNG allows chloroethylnitrosourea to produce DNA interstrand cross-links not observed in cells treated with chloroethylnitrosourea alone. *Carcinogenesis*, **4**, 759–763.

ZLOTOGORSKI, C. & ERICKSON, L.C. (1984). Pretreatment of human colon tumour cells with DNA methylating agents inhibits their ability to repair chloroethyl monoadducts. *Carcinogenesis*, **5**, 83–87.